# MiR-485-3p and miR-485-5p suppress breast cancer cell metastasis by inhibiting PGC-1*α* expression

**DOI:** 10.1038/cddis.2016.27

**Published:** 2016-03-24

**Authors:** C Lou, M Xiao, S Cheng, X Lu, S Jia, Y Ren, Z Li

**Affiliations:** 1 Department of Breast Surgery, The Affiliated Tumor Hospital, Harbin Medical University, Harbin, China

## Abstract

Breast cancer is the worldwide leading cause of cancer mortality in women. The majority of deaths from breast cancer arise from metastasis of local tumors. Cancer cells support their rapid proliferation by diverting metabolites into anabolic pathways, but during cancer metastasis, the proliferative program of invasive cancer cells is suspended for a migratory phenotype. In this study, we demonstrated that both mature forms of miRNA-485, miR-485-3p and miR-485-5p were involved in regulating mitochondrial respiration, cell migration and cell invasion in breast cancer cells by directly targeting and inhibiting the expression of PGC-1*α*. Specifically, the expression levels of both miR-485-3p and miR-485-5p were decreased in breast cancer tissues. Overexpression of miR-485-3p and miR-485-5p suppressed mitochondrial respiration and potential for cell migration and invasion *in vitro*, and also inhibited spontaneous metastasis of breast cancer cells *in vivo*. The suppression of mitochondrial respiration and cell invasion could be partially relieved by restoration of PGC-1*α* expression.

Breast cancer is one of the most commonly diagnosed malignant tumors, and is the leading cause of cancer mortality in women worldwide.^1^ A vast majority of deaths in breast cancer arise from metastasis, during which tumor cells proliferate, reorganize and migrate into the circulation, and consequently invade other tissues.^2^ Therefore, understanding the pathogenesis of breast cancer metastasis will facilitate the development of novel strategies for effective treatment of the disease.

In cancer cells, although the metabolic demands are primarily satisfied through the process of aerobic glycolysis, they support their rapid proliferation by diverting metabolites into anabolic pathways to accumulate cellular materials required for large-scale cell growth.^3, 4^ During cancer metastasis, the proliferative program of invasive cancer cells is suspended, and these cells acquire a migratory phenotype.^5^ The metabolic requirements for this transition process, however, are not well characterized. In a recent study, it was demonstrated that metastatic cancer cells specifically favored mitochondrial respiration and increased ATP production, and that they made use of the transcription co-activator, PGC-1*α*, to enhance oxidative phosphorylation, mitochondrial biogenesis and oxygen consumption rates. Loss of PGC-1*α* in cancer cells was shown to abolish their invasive potential and attenuate metastasis without affecting proliferation and tumor growth.^6^

Emerging evidence has suggested that in addition to protein-encoding genes, non-coding RNAs, especially microRNAs, also have important roles in the pathogenesis of cancer metastasis by regulating the expression of cancer-related genes.^7^ MicroRNAs (miRNAs) are small non-coding RNAs approximately 22 nucleotides in length. Although their detailed mechanism(s) of action is not clear, miRNAs are believed to act primarily in a post-transcriptional manner by binding to the 3′-untranslated region (3′-UTR) of target messenger RNA (mRNA), resulting in mRNA degradation or translational inhibition.^8^ Increasing numbers of studies have indicated that miRNAs are involved in regulating numerous cellular processes, including metabolic homeostasis, cell proliferation and cell apoptosis.^9^ Importantly, aberrant expression of miRNA has been reported in many cancer types, including prostate,^10^ colorectal^11^ and glioma.^12^ The roles miRNAs have in cancer onset, development and metastasis are complicated, because it has been noted that miRNAs can either act as oncogenes by repressing target mRNAs of tumor-suppressor genes or as tumor suppressors by negatively regulating oncogene mRNAs.^13^

In our study, we demonstrated for the first time that the miRNAs, miR-485-3p and miR-485-5p, are involved in regulating mitochondrial respiration, cell migration and cell invasion in breast cancer cells by directly targeting and inhibiting the expression of PGC-1*α*. Specifically, both miR-485-3p and miR-485-5p were downregulated in breast cancer tissues. Overexpression of miR-485-3p and miR-485-5p suppressed both mitochondrial respiration and potential for cell migration and invasion, and this suppression could be partially relieved by restoration of PGC-1*α* expression.

## Results

### The expression of miR-485-3p and miR-485-5p is downregulated in breast cancer

**Figure 1:**
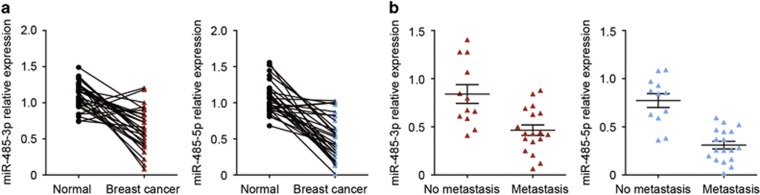
The expression of miR-485-3p and miR-485-5p is downregulated in breast cancer tissues. (**a**) The expression levels of miR-485-3p and miR-485-5p in cancer tissues compared with adjacent normal tissues in 30 breast cancer patients, *P*<0.001 by paired *t*-test. (**b**) The expression levels of miR-485-3p and miR-485-5p in breast cancer tissues with and without lymph node metastasis, *P*<0.001 by *t*-test

Although it has been suggested that miR-485 may exhibit some tumor-suppressor properties in a specific breast carcinoma cell line,^14^ the functional role of miR-485 in breast cancer development remained largely unclear. Therefore, we first sought to determine the levels of miR-485 expression in breast cancer tissues. We found that the expression levels of the mature forms of miR-485, miR-485-3p and miR485-5p were significantly downregulated in breast cancer tissues from 30 patients compared with adjacent normal tissues (Figure 1a). In addition, of the 30 breast cancer cases, there were 18 cases that had spread to lymph nodes. We then compared the expression levels of miR-485-3p and miR-485-5p between the breast cancer cases with and without lymph node metastasis, and determined that the expression levels of miR-485-3p and miR-485-5p were significantly reduced in breast cancer samples with lymph node metastasis (Figure 1b, Table 1). Taken together, these clinical data indicated that the expression levels of miR-485 were negatively correlated with development and metastasis potential of breast cancers, and therefore miR-485 could be involved in the molecular mechanism(s) of breast cancer progression.

### PGC-1*α* is a direct target of miR-485-3p and miR-485-5p

**Figure 2:**
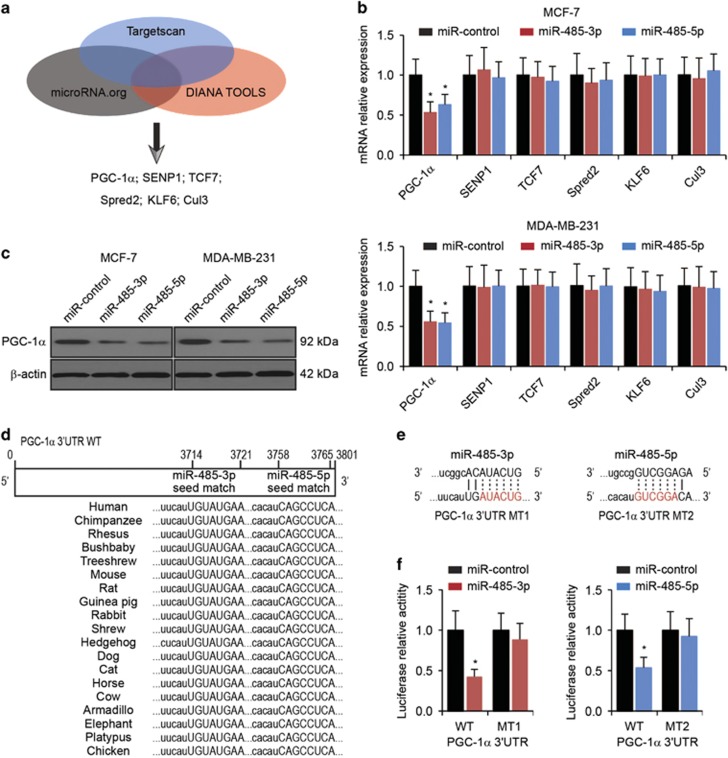
PGC-1*α* is a direct target of miR-485-3p and miR-485-5p. (**a**) Six predicted target genes (PGC-1*α*, SENP1, TCF7, Spred2, KLF6 and Cul3) of miR-485-3p and miR-485-5p by Targetscan, microRNA.org and DIANA TOOLS. (**b**) The expression levels of predicted target genes in MCF-7 and MDA-MB-231 cells with transfection of miR-control, miR-485-3p or miR-485-5p. *β*-Actin was used as an internal control. *P<0.05 by one-way ANOVA compared with miR-control-transfected cells. (**c**) Western blot analysis of PGC-1*α* in MCF-7 and MDA-MB-231 cells transfected with miR-control, miR-485-3p or miR-485-5p. *β*-Actin was used as loading control. *P<0.05 by one-way ANOVA compared with miR-control-transfected cells. (**d**) Schematic representation of the wild-type PGC-1*α* 3′-UTR (WT) showing putative miR-485-3p and miR-485-5p target sites. (**e**) Mutations of predicted binding sites of miR-485-3p (MT1) or miR-485-5p (MT2) in the PGC-1*α* 3′-UTR. (**f**) Relative luciferase activity in HEK293T cells co-transfected with WT or MT reporter plasmid and miR-control, miR-485-3p or miR-485-5p. *P<0.05 by *t*-test compared with miR-control-transfected cells

In many cases, miRNAs perform biological functions through negative regulation of their target genes.^15^ Using three public prediction algorithms, Targetscan, microRNA.org and DIANA TOOLS, we initially identified six genes (PGC-1*α*, SENP1, TCF7, Spred2, KLF6 and Cul3) predicted to be targets for miR-485-3p and miR-485-5p (Figure 2a). To validate the prediction, we transfected miR-control, miR-485-3p or miR-485-5p into MCF-7 and MDA-MB-231 cells, and determined the expression levels of the predicted genes by qRT-PCR. We found that miR-485-3p and miR-485-5p transfection led to significantly reduced expression of PGC-1*α* in both MCF-7 and MDA-MB-231 cells, but there were no significant changes in the expression levels of the other five predicted miR-485 target genes (Figure 2b). Furthermore, western blot analysis also confirmed that transfection of miR-485-3p and miR-485-5p resulted in decreased PGC-1*α* protein levels in both MCF-7 and MDA-MB-231 cells (Figure 2c).

We next identified potential miR-485-3p and miR-485-5p targeting sites in the 3′-UTR of PGC-1*α* (Figure 2d). To confirm the direct targeting sites of miR-485, we constructed reporter plasmids of the PGC-1*α* 3'-UTR containing either the wild-type or mutant forms of the potential miR-485-3p or miR-485-5p targeting sites (Figure 2e). Using the luciferase reporter assay, we found that miR-485-3p and miR-485-5p reduced the luciferase activity of the reporter plasmids containing their respective wild-type 3′-UTRs of PGC-1*α*. In contrast, miR-485-3p and miR-485-5p had no effect on the luciferase activity of the reporter plasmids containing their respective mutated 3′-UTRs of PGC-1*α* (Figure 2f). To elucidate PGC-1*α* expression in breast cancer samples and breast cancer tissues with lymph node metastasis, we performed qRT-PCR and found that PGC-1*α* was downregulated in breast cancer tissues (Supplementary Figure 1). A negative correlation was seen between miR-485 and PGC-1*α* (Supplementary Figure 2). Collectively, these results showed that miR-485-3p and miR-485-5p directly targeted the 3′-UTR of PGC-1*α* and negatively regulated PGC-1*α* expression.

### Both miR-485-3p and miR-485-5p regulate mitochondrial respiration, migration, invasion and proliferation of breast cancer cells

**Figure 3:**
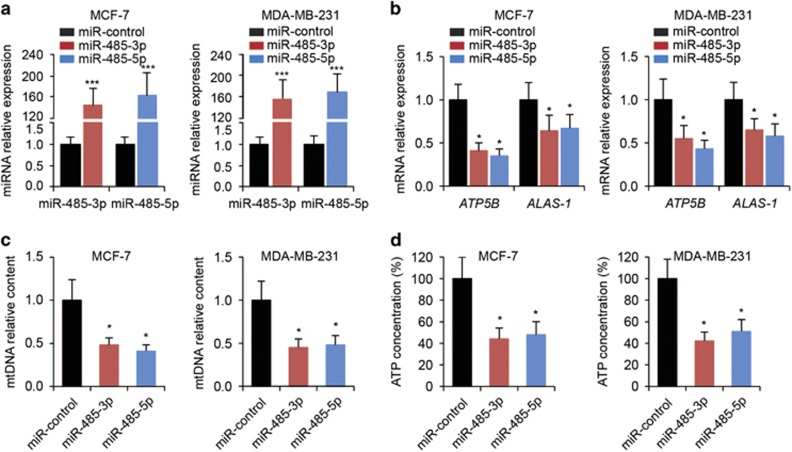
Reduction of mitochondrial respiration in breast cancer cells by miR-485-3p and miR-485-5p. (**a**) The expression levels of miR-485-3p or miR-485-5p in MCF-7 and MDA-MB-231 cells transfected with miR-control, miR-485-3p or miR-485-5p. ***P<0.001 by *t*-test compared with miR-control-transfected cells. (**b**) The expression levels of *ATP5B* and *ALAS-1* in MCF-7 and MDA-MB-231 cells transfected with miR-control, miR-485-3p or miR-485-5p. (**c**) Relative mtDNA content and (**d**) ATP concentrations in MCF-7 and MDA-MB-231 cells transfected with miR-control, miR-485-3p or miR-485-5p. *P<0.05 by one-way ANOVA compared with miR-control-transfected cells

It has recently been reported that PGC-1*α* is involved in regulating mitochondrial function and metastasis of breast cancer cells.^6^ As PGC-1*α* is a target for miR-485-3p and miR-485-5p, we first sought to investigate the functional roles of miR-485-3p and miR-485-5p in regulating mitochondrial respiration of breast cancer cells. We transfected miR-485-3p or miR-485-5p into MCF-7 and MDA-MB-231 breast cancer cells and confirmed overexpression (Figure 3a). We found that overexpression of miR-485-3p and miR-485-5p led to a significant reduction in the expression of two target genes of PGC-1*α*, *ATP5B* and *ALAS-1*, which have important roles in mitochondrial metabolism^16^ (Figure 3b). Furthermore, we examined the mitochondrial DNA (mtDNA) content and the ATP concentrations in MCF-7 and MDA-MB-231 with miR-485-3p or miR-485-5p overexpression, and found that both miR-485-3p and miR-485-5p significantly reduced the levels of mtDNA content (Figure 3c) and ATP concentrations (Figure 3d).

**Figure 4:**
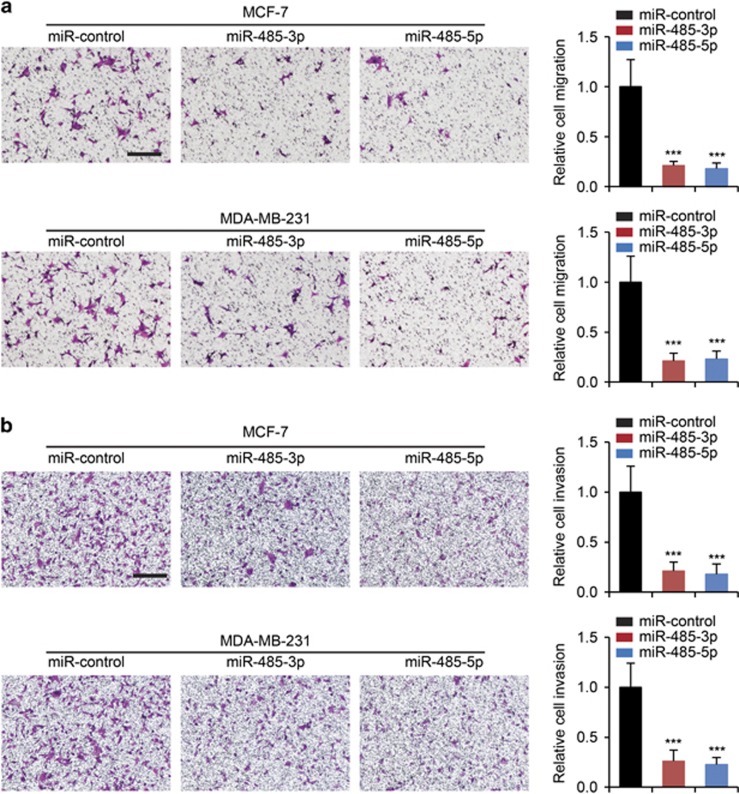
Inhibition of breast cancer cell migration and invasion by miR-485-3p and miR-485-5p. (**a**) Migration and (**b**) invasion assays in MCF-7 and MDA-MB-231 cells transfected with miR-control, miR-485-3p or miR-485-5p. Scale bars: 100 *μ*m. Cell migration and invasion results were normalized by cell proliferation results under the same treatment conditions. ***P<0.01 by one-way ANOVA compared with miR-control-transfected cells

**Figure 5:**
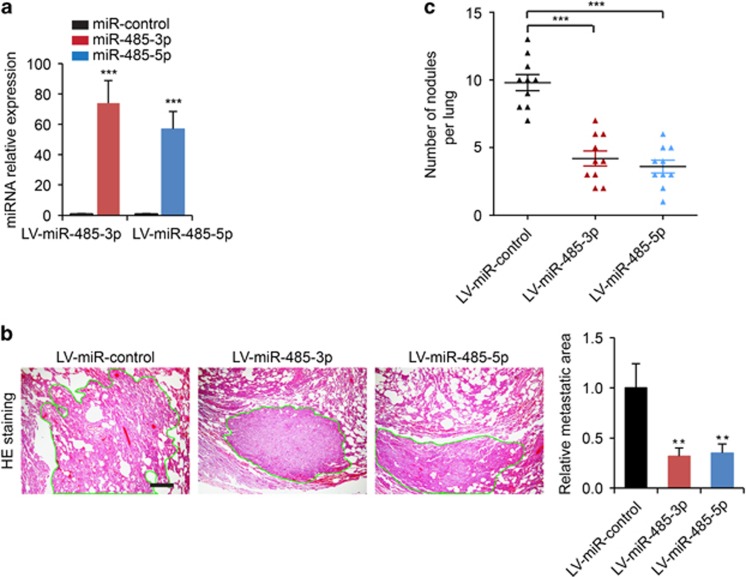
Inhibition of spontaneous lung metastatic capabilities of breast cancer cells by miR-485-3p and miR-485-5p. (**a**) The expression levels of miR-485-3p or miR-485-5p in MDA-MB-231 cells transduced with LV-miR-control, LV-miR-485-3p or LV-miR-485-5p. (**b**) Representative photos of H&E staining of lung tissue sections from mice subjected to tail vein injection of MDA-MB-231 cells stably expressing miR-control (LV-miR-control), miR-485-3p (LV-miR-485-3p) or miR-485-5p (LV-miR-485-5p), and quantification of relative metastatic lung surface area. (**c**) The quantification of metastatic nodules in lungs from mice subjected to tail vein injection of MDA-MB-231 cells stably expressing miR-control (LV-miR-control), miR-485-3p (LV-miR-485-3p) or miR-485-5p (LV-miR-485-5p). Scale bars: 200 *μ*m. **P<0.05, ***P<0.001 by one-way ANOVA compared with the LV-miR control group

As PGC-1*α* was also shown to have a pivotal role in the metastasis of breast cancer cells,^6^ we sought to determine whether miR-485-3p and miR-485-5p are able to regulate migration and invasion of breast cancer cells. We found that overexpression of miR-485-3p and miR-485-5p significantly inhibited both migration (Figure 4a) and invasion (Figure 4b) of MCF-7 and MDA-MB-231 breast cancer cells. In addition, we established MDA-MB-231 cells stably expressing miR-control, miR-485-3p and miR-485-5p (Figure 5a), and injected these cells into the tail veins of mice and examined the lung metastasis of the xenograft breast tumors. We determined that both miR-485-3p and miR-485-5p were able to reduce the metastatic area (Figure 5b) and the numbers of nodules in the lung (Figure 5c). To determine whether miR-485-3p and miR-485-5p affect breast cancer cell proliferation, the MTT assay and cell cycle analysis were performed and saw that both miR-485-3p and miR-485-5p suppressed cell proliferation and arrested more cells in the G0/G1 phases (Supplementary Figure 3). Taken together, these data indicated that miR-485-3p and miR-485-5p were involved in regulating mitochondrial respiration, metastasis and proliferation of breast cancer cells.

### Restoration of PGC-1*α* relieves the suppression of mitochondrial respiration, migration and invasion of breast cancer cells mediated by miR-485-3p and miR-485-5p

**Figure 6:**
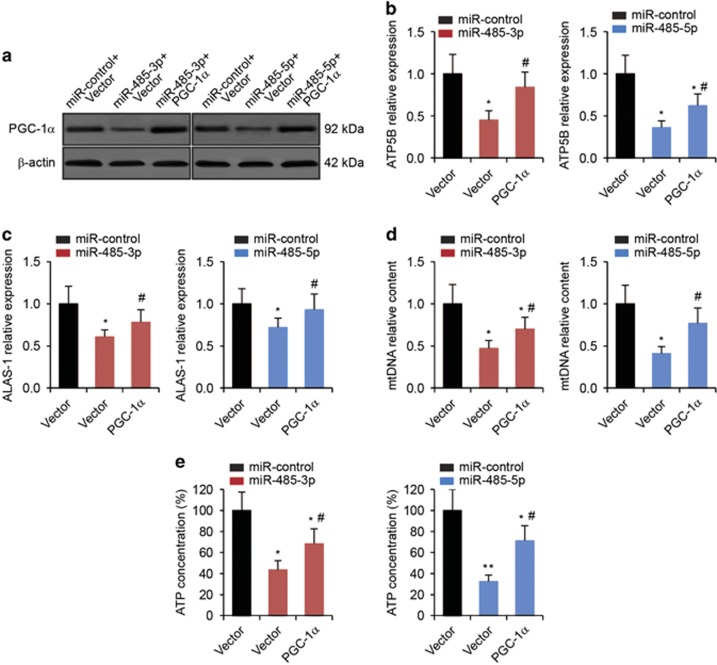
Restoration of PGC-1*α* reverses the suppression of mitochondrial respiration induced by miR-485-3p and miR-485-5p. (**a**) Western blot analysis in MCF-7 cells co-transfected miR-485-3p or miR-485-5p with either empty vector or PGC-1*α* plasmid. (**b**) The expression levels of *ATP5B* and (**c**) *ALAS-1* in MCF-7 cells as determined by qRT-PCR analysis. (**d**) Relative mtDNA content and (**e**) ATP concentrations in MCF-7 cells. *P<0.05, **P<0.01 compared with miR-control-transfected and empty vector-transfected cells by one-way ANOVA. ^#^*P*<0.05 compared with miRNA-transfected and empty vector-transfected cells by one-way ANOVA

**Figure 7:**
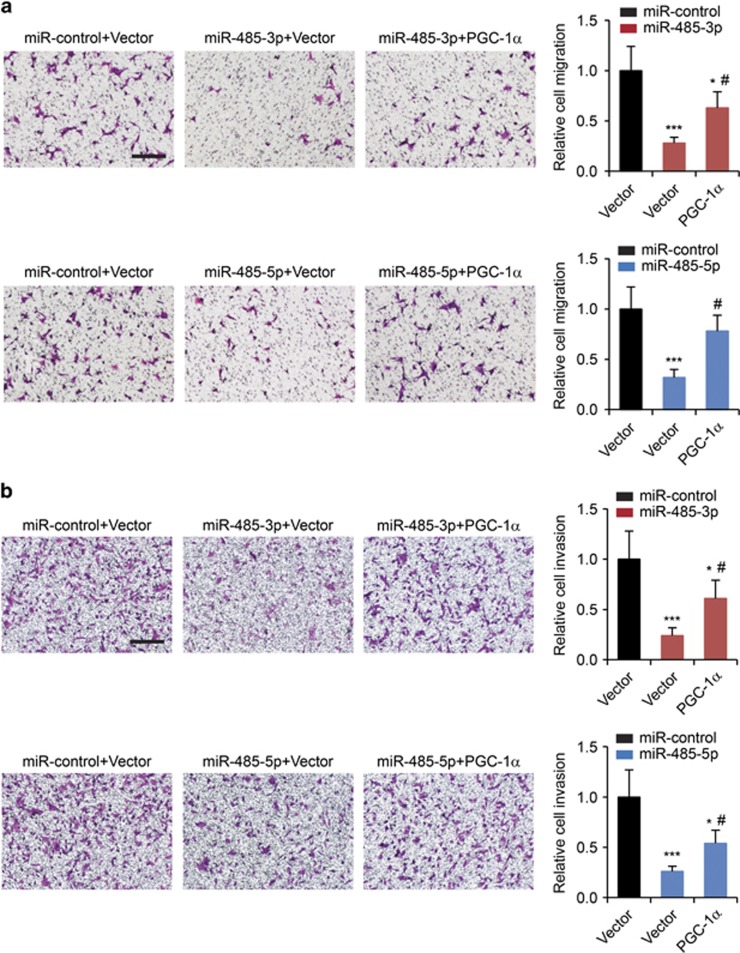
Restoration of PGC-1*α* relieves the inhibition of breast cancer cell migration and invasion induced by miR-485-3p and miR-485-5. (**a**) Migration and (**b**) invasion assays in MCF-7 cells co-transfected miR-485-3p or miR-485-5p with either empty vector or PGC-1*α* plasmid. Scale bars: 100 *μ*m. Cell migration and invasion results were normalized by cell proliferation results under the same treatment conditions. *P<0.05, ***P<0.001 compared with miR-control-transfected and empty vector-transfected cells by one-way ANOVA. ^#^*P*<0.05 compared with miRNA-transfected and empty vector-transfected cells by one-way ANOVA

If miR-485-3p and miR-485-5p suppress mitochondrial respiration, migration and invasion of breast cancer cells through targeting and inhibiting PGC-1*α* expression, restoration of PGC-1*α* expression should be able to relieve the suppression. To test this hypothesis, we transfected PGC-1*α* into MCF-7 cells and subjected them to miR-485-3p or miR-485-5p transfection. Transfection of PGC-1*α* evidently restored the protein levels of PGC-1*α* reduced by miR-485-3p or miR-485-5p transfection (Figure 6a). Significantly, the restoration of PGC-1*α* expression partially reversed the suppression of gene expression of *ATP5B* (Figure 6b) and *ALAS-1* (Figure 6c) induced by miR-485-3p or miR-485-5p overexpression. In addition, the restoration of PGC-1*α* expression also partially relieved the reduction of mtDNA content (Figure 6d) and ATP concentrations (Figure 6e) in MCF-7 cells with miR-485-3p or miR-485-5p overexpression. Furthermore, we found that restoration of PGC-1*α* expression in MCF-7 cells was also able to reverse the inhibition of cell migration (Figure 7a) and cell invasion (Figure 7b) caused by the overexpression of miR-485-3p and miR-485-5p. These results were consistent with the hypothesis that in breast cancer cells, miR-485-3p and miR-485-5p regulate mitochondrial respiration, cell migration and invasion, at least partially, by inhibiting the expression of PGC-1*α*.

## Discussion

In this study, we first demonstrated that the expression levels of the mature products of miRNA-485, miRNA-485-3p and miRNA-485-5p were significantly downregulated in breast cancer tissues, and that the expression levels of miRNA-485-3p and miRNA-485-5p were inversely correlated with the potential for metastatic dissemination in breast cancer patients. These results were consistent with recent findings that the expression levels of tumor-suppressing miRNAs were often downregulated in metastatic breast cancer. For example, miR-206 exhibited crucial tumor-suppressor roles in the progression of breast cancer by regulating cyclin-D2 expression.^17^ In addition, miR-720, which acts as a tumor suppressor by inhibiting cell migration and invasion, was found to be downregulated in primary breast carcinoma.^18^ The expression levels of miR-485 itself were also demonstrated by recent studies to be correlated with cancer risk and could serve as biomarkers for diagnosis of multiple types of cancers. It was reported that the expression of miR-485-5p was reduced in pediatric central nervous system tumors.^19^ In addition, there was a substantial correlation between miR-485 expression and pathological parameters in ovarian cancers,^20^ suggesting that miR-485 could be a reliable biomarker for ovarian cancers. Moreover, miR-485 has also been shown to mediate drug responsiveness in human leukemic lymphoblastic cells and rhabdomyosarcoma cells.^21^

Our study demonstrated that miR-485-3p and miR-485-5p were involved in regulating mitochondrial respiration, cell migration and cell invasion in breast cancer cells by directly targeting and inhibiting the expression of PGC-1*α*. PGC-1*α* has recently emerged as an important regulator of cancer metabolism, and as advanced therapeutic strategies are starting to focus on the unique metabolism patterns of cancer cells, PGC-1*α* has become a potential target for cancer therapy. In a recent study, it was shown that PGC-1*α*-mediated mitochondrial respiration in cancer cells that was functionally essential for metastasis. Migrating and invading cancer cells relied on PGC-1*α* to enhance mitochondrial respiration during their metastasis. Suppression of PGC-1*α* significantly impaired mitochondrial respiration and decreased the metastatic potential of cancer cells.^6^ Consistent with these findings, as PGC-1*α* is negatively regulated by miR-485-3p and miR-485-5p, overexpression of miR-485-3p and miR-485-5p significantly reduced mitochondrial respiration and cell migration and invasion in breast cancer cells.

The epigenetic relation between miR-485 and PGC-1*α* was confirmed by a rescue experiment. We replenished the miR-485-induced downregulation of PGC-1*α* by ectopic PGC-1*α* overexpression, and this manipulation partially reversed the phenotypes in mitochondrial respiration and cell migration and invasion induced by miR-485 overexpression. These findings indicated that the effects of miR-485-3p and miR-485-5p on mitochondrial respiration and cell migration and invasion occurred at least partially via a pathway involving PGC-1*α*. The fact that restoration of PGC-1*α* could not completely reverse the phenotypes induced by miR-485-3p and miR-485-5p suggested that there could be other signaling pathways downstream of miR-485-3p and miR-485-5p participating in the regulation of mitochondrial respiration and cell migration and invasion in breast cancer. Noticeably, a recent study showed that the miR-485-5p binding site, SNP rs8752, in the HPGD gene was associated with breast cancer risk.^22^

In addition, although miR-485-3p and miR-485-5p appeared to have a suppressive role in mitochondrial respiration and cell migration and invasion, their functions in other aspects of cancer development, such as cell growth and proliferation, are not understood. In this context, it is interesting to note that PGC-1*α*-mediated enhanced mitochondrial respiration and oxidative phosphorylation did not appear to impact upon glycolytic and anabolic rates, and did not affect cancer cell proliferation or the kinetics of primary tumor growth.^6^ Therefore, the roles of miR-485-3p and miR-485-5p in cancer cell growth and proliferation require further investigation.

## Materials and Methods

### Patients

A total of 30 patients with histologically confirmed breast cancer at Harbin Medical University Cancer Hospital were enrolled in this study from 2011 to 2012. All patients underwent surgical resection without prior radiotherapy or chemotherapy. Primary breast tumors, paired adjacent normal breast tissue (breast tissue >3 cm from the tumor) and lymph nodes were collected from each patient. Tissue samples were collected immediately, snap frozen in liquid nitrogen and stored at −80 °C for analysis. Informed consent was obtained from all participants. The study was approved by the institutional review board at Harbin Medical University.

### Cell culture and transfection

Breast cancer cell lines (MCF-7 and MDA-MB-231) and 293 T cells were purchased from the Cell Bank of Type Culture Collection of the Chinese Academy of Sciences, Shanghai, China. The cells were cultured in high-glucose Dulbecco's modified Eagle's medium (DMEM; Gibco, Grand Island, NY, USA) containing 10% heat-inactivated fetal bovine serum (FBS; Gibco), 100 U/ml penicillin, 100 U/ml streptomycin and 2 mmol/l l-glutamine, at 37 °C in a humidified 5% CO_2_ atmosphere.

Plasmids and miRNA were transfected in antibiotic-free Opti-MEM medium (Life Technologies, Carlsbad, CA, USA) with lipofectamine 2000 reagent (Life Technologies) according to the manufacturer's instructions. The full-length cDNA of PGC-1*α* without the 3′-UTR region was amplified from human genomic DNA and cloned into pcDNA to generate the pcDNA-PGC-1*α* constructs. All functional assays were performed 24 h after transfection.

### Western blot analysis

Total proteins of cultured cells were extracted using RIPA buffer (50 mM Tris HCl, 137 mM NaCl, 0.5% Na deoxycholate, 1% Triton X-100, 0.1% SDS, protease inhibitors) at 4 °C for 30 min. The cell lysates were centrifuged at 4 °C for 10 min at 12 000 *g* to separate soluble proteins, and the protein concentrations were determined using a bicinchoninic acid protein assay kit (Tiangen Biotech Co., Beijing, China). Proteins were resolved by 10% SDS-PAGE and transferred onto a nitrocellulose membrane. The membrane was blocked with 5% (w/v) non-fat powdered milk in TBS containing 0.1% Tween for 1 h, washed with TBS/Tween, incubated overnight at 4 °C with antibodies against PGC-1*α* and *β*-actin (Santa Cruz Biotechnology, Dallas, TX, USA), and incubated for 2 h with appropriate secondary antibodies. Signals were visualized by chemiluminescence (Beyotime, Beijing, China).

### qRT-PCR

Total RNA was extracted from breast cancer cells and tissues with the TRIzol RNA isolation system (Life Technologies). For miRNA expression, qRT-PCR was performed using TaqMan miRNA assays with specific primer sets (Applied Biosystems, Foster City, CA, USA) according to the manufacturer's protocols. The PrimeScript One-step RT-PCR Kit (Takara, Dalian, Liaoning, China) was used for mRNA amplification.

The primers used were as follows: *ATP5B*: forward primer 5′-TCACCCAGGCTGGTTCAGA-3′, reverse primer 5′-AGTGGCCAGGGTAGGCTGAT-3′ *ALAS-1*: forward primer 5′-GGCAGCACAGATGAATCAGAGAG-3′, reverse primer 5′-TTCAGCAACCTCTTTCCTCACGG-3′ *PGC-1α*: forward primer 5′-AACAGCAGCAGAGACAAATGCACC-3′, reverse primer 5′-TGCAGTTCCAGAGAGTTCCACACT-3′ *SENP1*: forward primer 5′-AAGATTCCCAGACTCCAACTCCCA-3′, reverse primer 5′-TGAATGTTCCCGCTCCTGCAAT-3′ *TCF7*: forward primer 5′-CGTTCCTTCCGATCAGT-3′, reverse primer 5′-AGGGCTAGTAGGCAGTTCTGTG-3′ *Spred2*: forward primer 5′-GAAGGTACCCGGACAGGAAGATGAAGGG-3′, reverse primer 5′-GAACTCGAGAGGGAGAAGGGAGGGAAACT-3′ *KLF6*: forward primer 5′-CTGCCGTCTCTGGAGGAGT-3′, reverse primer 5′-TCCACAGATCTTCCTGGCTGTC-3′ *Cul3*: forward primer 5′-GCCATGGTGATGATTAGAGACA-3′, reverse primer 5′-CCGTACCAACTTGATCTCGAAAA-3′ *β-*actin: forward primer 5′-GGCTGTATTCCCCTCCATCG-3′, reverse primer 5′-CCAGTTGGTAACAATGCCATG-3′.

### Luciferase assays

The wild-type (WT) 3′-UTRs of human PGC-1*α* and a mutated (MT) 3′-UTR of human PGC-1*α* containing mutations in the predicted miR-485 seed regions were amplified and subcloned into the psiCHECK-2 luciferase reporter vector (Promega, Madison, WI, USA). For the luciferase activity assay, 293 T cells were plated into 24-well plates and transfected with psiCHECK-2-PGC-1*α*-3′UTR-WT or psiCHECK-2-PGC-1*α*-3′UTR-MT, with miR-control, miR-485-3p or miR-485-5p. A Renilla luciferase vector (Promega) was co-transfected to normalize for transfection efficiency. After 48 h of transfection, firefly and Renilla luciferase activity was determined by the Dual-Luciferase Reporter Assay System (Promega). Relative firefly luciferase activity was measured by normalizing to the Renilla luciferase activity.

### Determination of mtDNA content

Total genomic DNA was extracted from breast cancer cells, using the Qiagen DNeasy Kit (Qiagen, Valencia, CA, USA) according to the manufacturer's protocol. The quality of DNA was determined using the Nanodrop spectrophotometer (ThermoFisher Scientific, Rockford, IL, USA). To quantify mtDNA copy numbers in peripheral blood, qRT-PCR was performed using SYBR Green reagents. The amplification of DNA content was obtained using specific primers for a mitochondria encoded gene (ND1). A nuclear encoded gene (HGB) was also quantified and used as control. The amplification of the genes and the level of mtDNA content were determined by qRT-PCR. The primers used were as follows: *ND1*: forward primer 5′-CCCTAAAACCCGCCACATCT-3′, reverse primer 5′-GAGCGATGGTGAGAGCTAAGGT-3′ *HGB*: forward primer 5′-GTGCACCTGACTCCTGAGGAGA-3′, reverse primer 5′-CCTTGATACCAACCTGCCCAG-3′.

### Determination of cellular ATP concentrations

Cellular ATP concentrations were measured in breast cancer cells using a bioluminescence assay kit (Promega) according to the manufacturer's instructions. Briefly, the reaction generated signals proportionate to ATP content. The bioluminescence was measured using a microplate multimode reader (Promega).

### Migration and invasion assays

Migration and invasion assays were performed using 8-*μ*m pore size transwell inserts (BD Biosciences, San Jose, CA, USA). In the migration assay, cells (1 × 10^5^) in 0.2 ml of serum-free medium were added to the upper chambers, and 0.6 ml of DMEM containing 10% FBS was added to the bottom chambers. Cells were then cultured for 8 h in a 37 °C incubator. For the invasion assay, diluted Matrigel (BD Biosciences) was used to coat the insert chambers' membranes. Cells were cultured for 24 h under the same conditions. Cells that migrated into or invaded the bottom chambers were fixed with paraformaldehyde, stained with crystal violet and quantified in six random fields.

### Xenograft model

MDA-MB-231 cells stably expressing miR-control, miR-485-3p or miR-485-5p were established as previously described.^23^ Human miR-485-3p and miR-485-5p were cloned into the lentivirus expression vector pLenti6.3/V5-DEST to generate pLenti6.3/V5-DEST–miR-485-3p and pLenti6.3/V5-DEST–miR-485-5p. MiR-485-3p, miR-485-5p expression constructs, or control vector were packaged with ViraPower Packaging Mix (ThermoFisher Scientific) in the 293FT packaging cell line and concentrated 48 h after transfection. MDA-MB-231 cells were infected with control, LV-miR-485-3p or LV-miR-485-5p, and selected by blasticidin. The expression of miR-485-3p or miR-485-5p was determined by real-time PCR.

Animal experiments were performed according to the Guide for the Care and Use of Laboratory Animals of Harbin Medical University. The protocol was approved by the Institutional Animal Care and Use Committee of Harbin Medical University. All surgeries were performed under sodium pentobarbital anesthesia, and all efforts were made to minimize suffering. MDA-MB-231 cells (1.5 × 10^6^) were suspended in 200 *μ*l of PBS and injected into the tail vein of each BALB/c male nude mouse (6–7 weeks old; 10 mice per group). At day 80 after cell injection, all mice were killed after anesthetic overdose, and their lungs were removed and examined. The nodules in the lung were quantified and subjected to hematoxylin and eosin (H&E) staining.

### Statistics

All experiments were performed at least three times. Data were expressed as mean±S.D. Statistical significance was analyzed using GraphPad Prism 5.0 (GraphPad Software, Inc., La Jolla, CA, USA). One-way analysis of variance (ANOVA) was performed for serial analysis, whereas two treatment groups were compared by the unpaired Student's *t*-test. Differences were considered statistically significant when *P*-value was <0.05.

## Figures and Tables

**Table 1:** Analysis of the correlation between miR-485-3p, miR-485-5p and clinicopathologic parameters in breast cancer

**Viable**	**Cases**	**miR-485-3p**			**miR-485-5p**		
		**Low**	**High**	***P*-value**	**Low**	**High**	***P*-value**
*Age, years*							
<50	17	7	10	0.231	9	8	0.500
⩾50	13	8	5		6	7	

*Tumor size, cm*							
<2	9	5	4	0.500	5	4	0.500
⩾2	21	10	11		10	11	

*Stage*							
I–II	14	4	10	0.033	3	11	0.005
III–IV	16	11	5		12	4	

*Lymph node*							
No metastasis	12	3	9	0.030	2	10	0.004
Metastasis	18	12	6		13	5	

## Abbreviations

microRNA: miR/miRNA
mRNA: messenger RNA
3′-UTR: 3′-untranslated region
DMEM: Dulbecco's modified Eagle's medium
FBS: fetal bovine serum
WT: wild type
MT: mutated
LV: lentivirus
H&E: hematoxylin and eosin
ANOVA: analysis of variance
mtDNA: mitochondrial DNA
