# Supplementary figures and images for: MiR-485-3p and miR-485-5p suppress breast cancer cell metastasis by inhibiting PGC-1α expression
Source: Cell Death Dis. 2016 Mar 24;7(3):e2159–. doi: 10.1038/cddis.2016.27 (PMC4823935; doi:10.1038/cddis.2016.27)

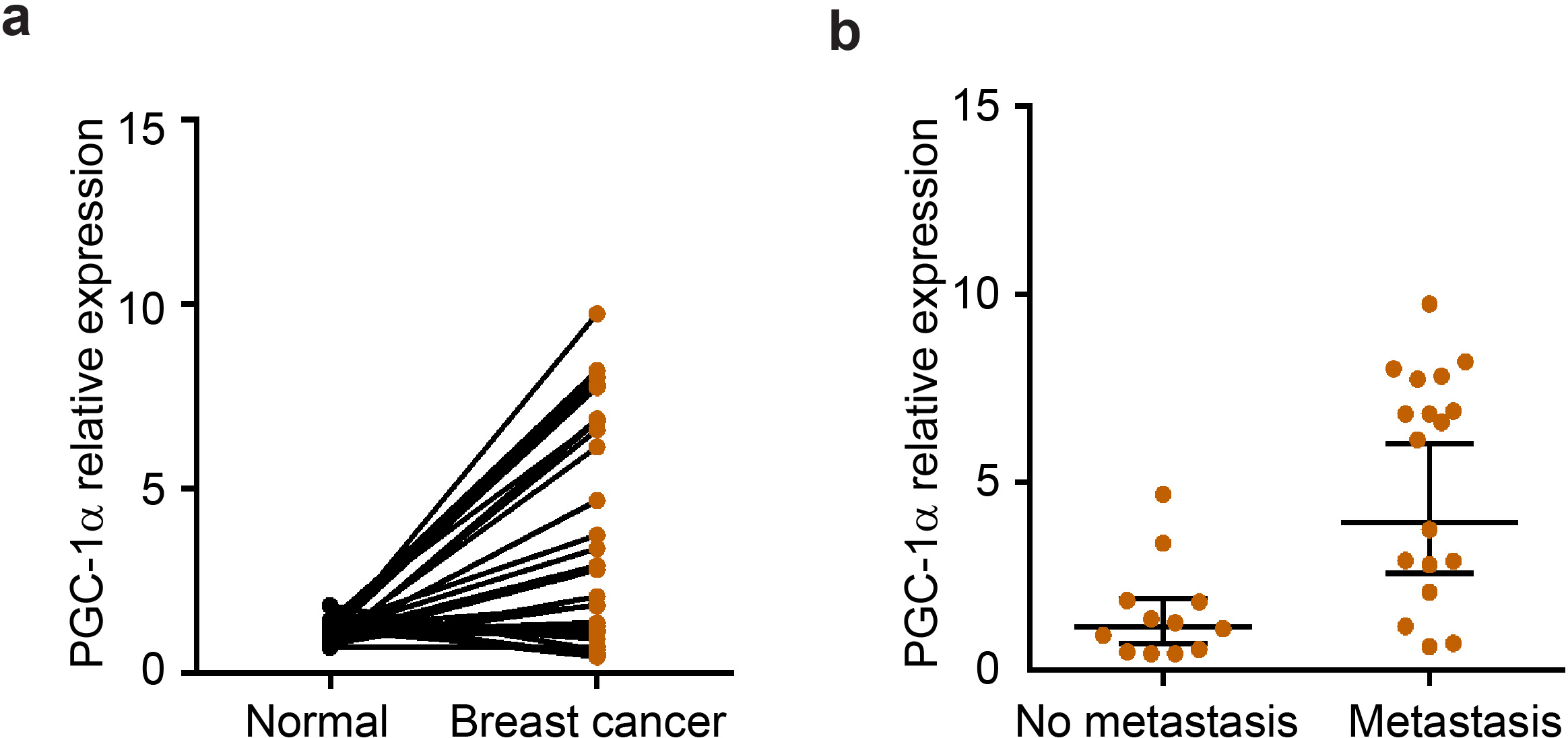

Supplement: Supplementary Figure 1 [file cddis201627x1.tif]

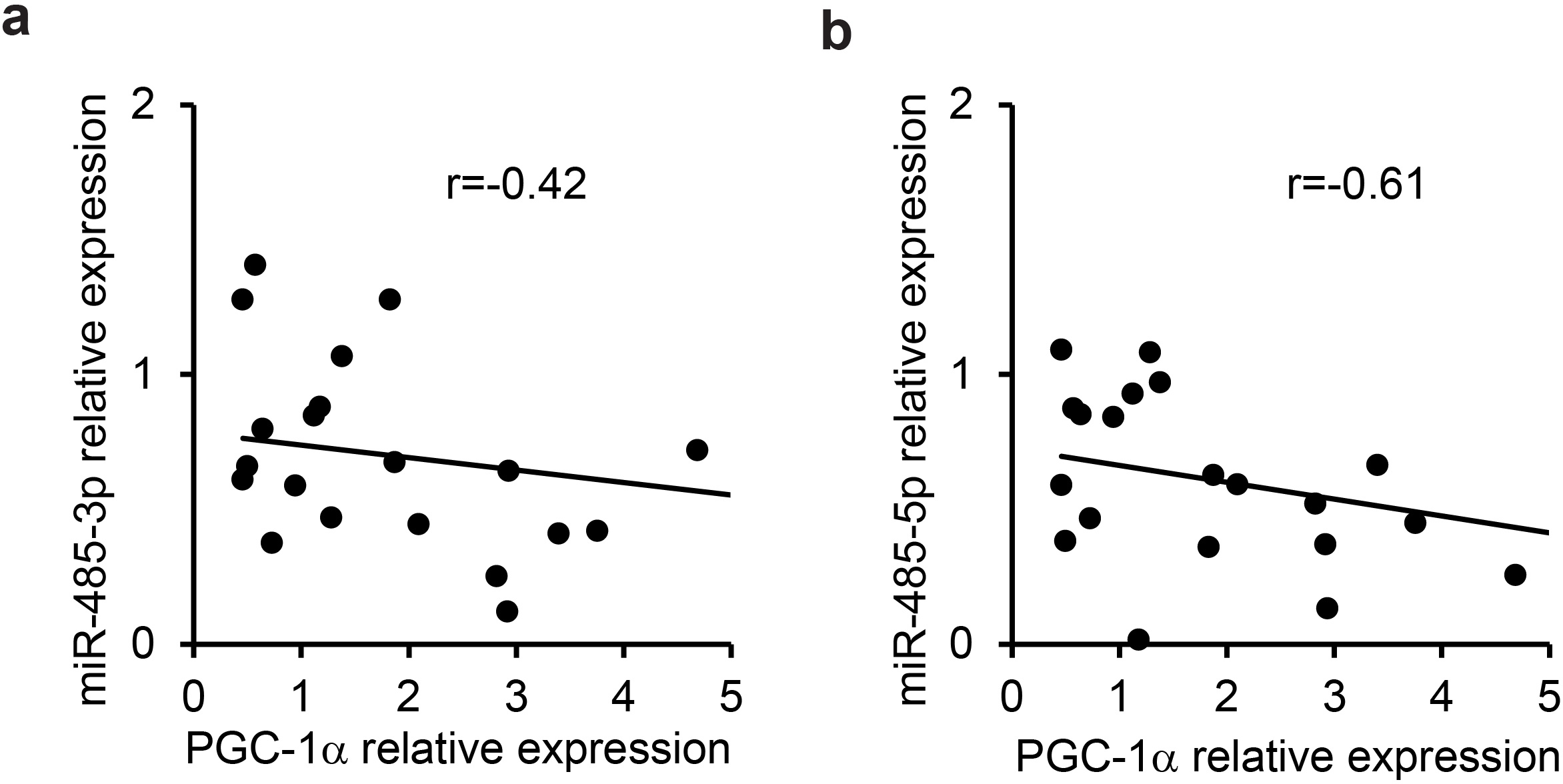

Supplement: Supplementary Figure 2 [file cddis201627x2.tif]

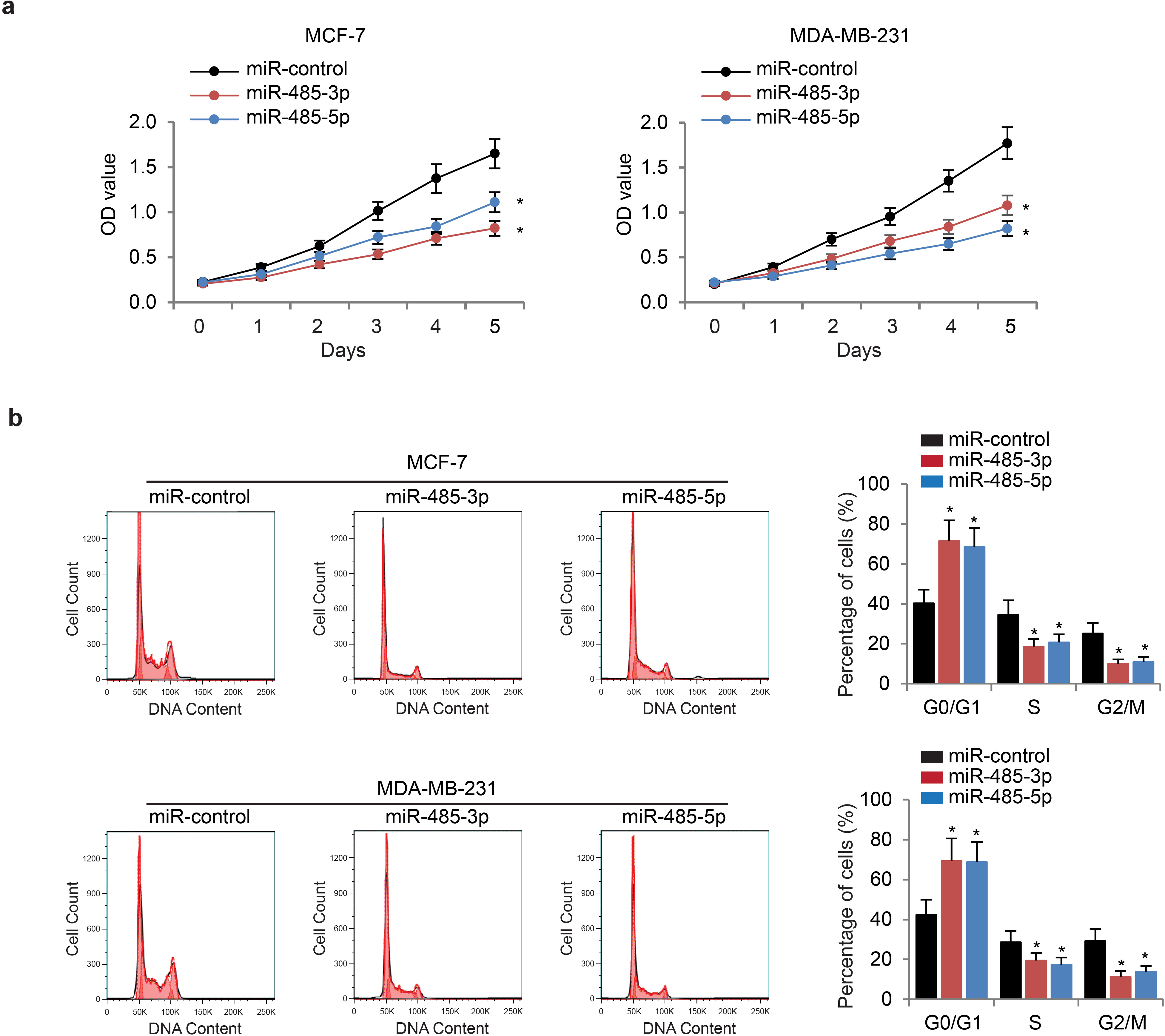

Supplement: Supplementary Figure 3 [file cddis201627x3.tif]
