# Supplementary material for: MiR-485-3p and miR-485-5p suppress breast cancer cell metastasis by inhibiting PGC-1α expression
Source: Cell Death Dis. 2016 Mar 24;7(3):e2159–. doi: 10.1038/cddis.2016.27 (PMC4823935; doi:10.1038/cddis.2016.27)
Supplement: Supplementary Figure Legends [file cddis201627x4.docx]

**Supplemental Figure 1.** The expression of PGC-1α is upregulated in breast cancer tissues

(a) The expression levels of PGC-1α in cancer tissues compared to adjacent normal tissues in 30 breast cancer patients, *P*<0.001 by paired *t*-test. (b) The expression levels of PGC‑1α in breast cancer tissues with and without lymph node metastasis, *P*<0.001 by paired *t*-test.

**Supplemental Figure 2.** Negative correlation between miR-485 and PGC-1α expression in breast cancer tissues (a) Pearson’s correlation between the expression of miR-485-3p and PGC‑1α (r=-0.42, *P*<0.05 by *t*-test). (b) Pearson’s correlation between the expression of miR‑485-5p and PGC-1α (r=-0.61, *P*<0.05 by *t*-test).

**Supplemental Figure 3.** miR-485-3p and miR-485-5p suppress breast cancer cell proliferation

(a) MTT and (b) cell cycle analysis in MCF-7 and MDA-MB-231 cells transfected with miR-control, miR-485-3p or miR-485-5p. **P*<0.05 compared to miR-control-transfected cells by one-way ANOVA.
